# Evaluation of Spontaneous Overtime Methemoglobin Formation in Post-Mortem Blood Samples from Real Cases in Critical Storage Conditions

**DOI:** 10.3390/toxics12090670

**Published:** 2024-09-14

**Authors:** Sara Gariglio, Maria Chiara David, Alessandro Mattia, Francesca Consalvo, Matteo Scopetti, Martina Padovano, Stefano D’Errico, Donato Morena, Paola Frati, Alessandro Santurro, Vittorio Fineschi

**Affiliations:** 1 DIFAR—Department of Pharmacy, University of Genova, 16132 Genova, Italy; sara.gariglio@edu.unige.it; 2 Department of Public Security, Health Central Directorate, Research Center and Forensic Toxicology Laboratory, Ministry of the Interior, 00184 Rome, Italy; mariachiara.david@gmail.com (M.C.D.); alessandro.mattia@poliziadistato.it (A.M.); 3 Department of Medicine, Surgery and Dentistry, Schola Medica Salernitana, University of Salerno, 84081 Baronissi, Italy; fconsalvo@unisa.it; 4 Department of Medical Surgical Sciences and Translational Medicine, Sapienza University of Rome, 00189 Rome, Italy; matteo.scopetti@uniroma1.it; 5 Department of Anatomical, Histological, Forensic and Orthopedic Sciences, Sapienza University of Rome, 00161 Rome, Italy; martina.padovano@uniroma1.it (M.P.); donato.morena@uniroma1.it (D.M.); paola.frati@uniroma1.it (P.F.); 6 Department of Medicine, Surgery, and Health, University of Trieste, 34137 Trieste, Italy; sderrico@units.it

**Keywords:** methemoglobin, nitrites, nitrates, suicide, storage, sample degradation, post-mortem investigation, spectrophotometry, forensic toxicology

## Abstract

Nitrite/nitrate poisoning is an emerging problem, with an ongoing escalation of reported self-administration with suicidal intent in several countries. Nitrites toxicity mainly consists of their interaction with hemoglobin (Hb), causing its oxidization to methemoglobin (MetHb). In order to give support to the correct procedures for the analysis of these cases, this study aims to evaluate spontaneous sample degradation and consequent MetHb formation in the typical storage conditions of a forensic toxicology laboratory. Two different types of samples have been used in this study: the first stage of our study consisted of a retrospective analysis of blood samples obtained by judicial autopsies already stored in the toxicology laboratory, collected over four years (2018–2021), while the samples used for the second stage were appositely collected during judicial autopsies. The data obtained by the application of a derivative spectrophotometry method on these samples suggest that there seems not to be a maximum threshold for MetHb formation within which it is possible to state with a sufficient grade of certainty that the concentration of MetHb found is consistent with an ante-mortem formation and is not the result of an artifact due to sample degradation and storage conditions. On the other hand, the results suggest that MetHb formation depends on the time passed between sample collection and analysis, so that a tempestive sample processing, performed as soon as the samples are received in the laboratory, is crucial to obtain the maximum reliability and diagnostic values from the data when MetHb quantitation is necessary.

## 1. Introduction

Over the years, forensic pathology has registered the spread of new methods of suicide, such as the ingestion of nitrites and nitrates, especially involving teenagers and young adults [1,2]. This relatively unusual occurrence has been reported with increasing frequency in the news media and in recent scientific publications, where several authors have warned of the appearance of intoxications caused by suicide using this compound, and cases have been recorded in Canada, South Korea, the United States, and Portugal. Overall, it emerges that the problem of nitrite/nitrate poisoning is quite common worldwide, with an ongoing escalation in reported self-administration with suicidal intent in several countries [3,4,5,6,7,8,9,10,11,12,13].

Potassium and sodium nitrates (KNO_3_ and NaNO_3_) and nitrites (KNO_2_ and NaNO_2_) are white or slightly yellow odorless inorganic salts. While nitrates are mainly used as fertilizers and food additives, as well as in the manufacturing process of gunpowder, nitrites are commonly used in the conservation process of cured meats, sausages, meat, and fresh fish like tuna fish, being antimicrobic agents and helping to improve products’ look and color.

Nitrites have higher toxicity for human beings compared to nitrates, causing severe intoxication and hypoxia. Nitrites toxicity mainly consists of their interaction with hemoglobin (Hb), causing its oxidization to methemoglobin (MetHb) [14]. Methemoglobin, thus obtained, is unable to bind and transport oxygen, therefore causing progressive hypoxia and death from functional anemia and development of lactic acidosis. A blood sample with an elevated MetHb concentration is typically characterized by a “brown chocolate” color [5]. This discoloration may also be observed in certain organs, including the heart and the kidney [4]. Nitrates can also be dangerous since they can be converted into nitrites in the oral cavity and bowel [15,16,17].

The path of MetHb formation depends on the form of Hb with which nitrite ions interact. Indeed, the reaction of the nitrite ion with the oxygenated form of Hb follows a complex autocatalytic mechanism, whose final product is the double oxidization of nitrite to nitrate and of Hb to MetHb. On the other hand, the reaction of nitrite with deoxygenated Hb causes its oxidization to MetHb and the reduction of nitrite ion to nitrogen monoxide (NO), which bonds to Hb forming nitrosyl-hemoglobin. Excluding cases in which oxygen saturation is extremely low, MetHb is the main product of this biochemical reaction [18,19].

The physiological concentration of MetHb is normally below 2%. Although methemoglobinemia levels under 20% are typically asymptomatic, when MetHb levels reach 10–20%, the characteristic “brown chocolate” color of the blood becomes noticeable [20]. Hypoxia’s symptoms start to become evident when MetHb reaches 20–35%, and for MetHb 20–50% dyspnea, tachypnoea, tachycardia, fatigue, confusion, and vomiting usually occur. A blood concentration of MetHb 50–70% can cause loss of conscience and death, and methemoglobinemia above 70% is usually reported as lethal. However, there is an important interindividual variability in MetHb tolerance, since MetHb-related deaths are also observed in the case of significantly inferior values (~33%) [4]. At the same time, a case of a patient who survived despite a 94% value of MetHb (tempestively treated with methylene blue) is described in the international literature [21].

The chemical-toxicological assessment of the cause of death from nitrite/nitrate poisoning is usually challenging since it is uncommon for forensic toxicology laboratories to have an available or sufficiently validated and robust method for direct analysis of nitrates for forensic purposes [2,22,23]. Therefore, the determination of MetHb based on spectrophotometry, despite being an indirect method, is considered the gold standard analytical method for indicating a death from nitrites/nitrates poisoning [24,25].

One of the biggest challenges in MetHb assessment is the fact that it can be both decreased by MetHb reductase and microbial activity in post-mortem samples [26], and spontaneously produced in post-mortem specimens due to spontaneous oxidization, sample degradation, and light exposition, processes which are all influenced by storage conditions. Even though some studies have investigated the best conditions for the storage of blood samples on which MetHb analysis is needed [26,27,28,29], little has been done to evaluate the maximum threshold for the spontaneous formation of post-mortem MetHb in real blood samples stored in the typical conditions of a forensic laboratory (i.e., at –20 °C, without any preservative, with the possibility of light- and air-exposure and several cycles of freezing and unfreezing).

Such a problem seems to be crucial if we consider that the analysis of MetHb concentration post-mortem is a non-routinary activity, frequently assessed as unnecessary immediately after death, especially when solid evidence that nitrite/nitrate poisoning may be involved in the cause of death is missing. As a result, samples are often collected without special precautions, and the analysis of MetHb may be requested even months after collection. These samples may have undergone multiple freeze–thaw cycles, been exposed to light, and not stored under the recommended conditions to minimize MetHb formation.

Therefore, this study aims to evaluate the spontaneous degradation of samples and the subsequent formation of MetHb under typical storage conditions found in a forensic toxicology laboratory. Specifically, it examines samples stored in a freezer without any preservatives and subjected to multiple freeze-thaw cycles. The objective is to determine the maximum period within which it can be confidently stated that the concentration of MetHb detected in blood samples reflects ante-mortem formation and is not an artifact resulting from sample degradation and storage conditions.

## 2. Materials and Methods

### 2.1. Samples

The determination was performed with a fast and easy spectrophotometric method on samples obtained from judicial autopsy cases and stored in critical conditions. The first stage of our study consisted of a retrospective analysis of 95 blood samples already stored in the toxicology laboratory, obtained by judicial autopsies ordered by a prosecutor and necessarily performed at variable and different post-mortem intervals.

In particular, these samples were taken from real cases handled by a forensic toxicology laboratory over time. They had already undergone forensic analysis and were no longer needed for further investigation. These samples were stored under varying, potentially critical conditions and subjected to different, non-standardized freeze–thaw cycles as part of various analytical activities.

These samples were collected over four years: 2018 (n = 20), 2019 (n = 19), 2020 (n = 20), and 2021 (n = 36).

The samples used in the second stage of the study were additional blood samples specifically collected during judicial autopsies.

All samples were collected from autopsies in which the cause of death was known and not related to events that may produce a biological increase in MetHb ante-mortem. In addition, all samples used for the study were screened for exogenous substances with a GC-MS method at the time of admission, and the samples in which were detected the most common drugs known to cause methemoglobinemia (i.e., nitrates/nitrites, phenacetin, dapsone, primaquine, chloroquine, hydroxylamine, zopiclone, sulfonamides such as sulfamethoxazole, and local anesthetics such as benzocaine, prilocaine, and cetacaine) were excluded from the data set as well.

All samples were stored in 20 mL HDPE scintillation vials (Aptaca 1221, Canelli, Italy) since collection, with no preservatives added to enhance MetHb stability. The samples were kept at −20 °C from the time of collection and had undergone at least two freeze–thaw cycles before analysis.

All samples were analyzed between February and May 2022.

### 2.2. Materials and Equipment

A UV-visible spectrophotometer (Fulltech Instruments V-1600 Visible Spectrophotometer, Rome, Italy) with quartz 3 mL cuvettes was used for the analysis. Deionized water and potassium ferricyanide were used (Sigma-Aldrich, Milan, Italy). All volumetric laboratory glassware was calibrated before use. Data collection and elaboration were performed on Excel^®^ 2021 calculation worksheets.

### 2.3. Procedure

The procedure was based on the derivative spectrophotometry method described by Cruz-Landeira et al. [30], adapted to the features of the UV-visible spectrophotometer used in this study.

### 2.4. Solutions’ Preparation

For the analysis, two different solutions were prepared from each sample.

Solution A: 100 μL of blood was added through a volumetric micropipette (Eppendorf, Milan, Italy) into a 10 mL Pyrex^®^ volumetric flask (Sigma-Aldrich, Milan, Italy), half full of deionized water. The volumetric flask was filled until reaching the calibration mark, then closed with a tight-fitting stopper, and mixed by inverting it several times until a homogeneous solution was obtained. An appropriate aliquot of this solution was then transferred into a quartz cuvette and placed in the UV-Vis spectrophotometer. An identical cuvette was filled with deionized water and used as blank A for solution A.

Solution B: 100 μL of blood was added through a volumetric micropipette (Eppendorf, Milan, Italy) into a 10 mL Pyrex^®^ volumetric flask (Sigma-Aldrich, Milan, Italy), half full with deionized water. The Hb present in the blood was then totally oxidized to MetHb using an excess aqueous solution of potassium ferricyanide (K_3_[Fe(CN)_6_]). Specifically, 50 μL of a 0.1 mg/μL potassium ferricyanide solution was added to water and blood solution. The volumetric flask was filled until reaching the calibration mark, then closed with a tight-fitting stopper, and mixed by inverting it several times until a homogeneous solution was obtained. An adequate aliquot of the thus obtained solution was then transferred into a quartz cuvette and placed in the UV-Vis instrument. A blank B for solution B was prepared to consider the effect of the oxidizing agent on the absorbance value. For this solution, 50 μL of a 0.1 mg/μL potassium ferricyanide solution was added to a 10 mL Pyrex^®^ volumetric flask (Sigma-Aldrich, Milan, Italy), half full with deionized water. The volumetric flask was filled until reaching the calibration mark, then closed with a tight-fitting stopper, and mixed by inverting it several times until a homogeneous solution was obtained. An adequate aliquot of the thus obtained solution was then transferred into a cuvette identical to the one used for solution B and placed in the UV-Vis instrument.

### 2.5. Instrumental Parameters

The instrument was used in “multiwavelength” mode, and eight wavelengths were set to read the absorbance of the various solutions. Those wavelengths were: 620 nm, 625 nm, 630 nm, 635 nm, 640 nm, 645 nm, 650 nm, and 655 nm. In particular, these wavelengths were specially and directly selected to quantitate MetHb, in consideration of absorbance spectra previously reported, among others, in the standard procedure by Cruz-Landeira et al., where MetHb is described to absorb at ~645 nm, with a positive peak that is not affected by the other interfering components [30]. The instrument was first zeroed with blank A solution, then the absorbance values for solution A were registered. The third step was to zero the instrument again with a blank B solution and then read the absorbance values for solution B. Those values were also registered for data analysis.

### 2.6. Mathematical Data Analysis

Since the spectrophotometer used for this study was not equipped with a “scan” mode, and, therefore, it did not allow to obtain the absorbance spectrum of the two solutions directly nor to register the derivative spectrum directly, the use of an interpolating equation was required. Therefore, the eight absorbance values for both solution A and solution B were analyzed with a dispersion graph and interpolated with a fourth-degree polynomial least square interpolation algorithm to obtain an accurate approximation of the absorbance spectrum in the analyzed region, obtaining for each solution an interpolating polynomial in the form of the following equation:

(1) $$y=ax^{4}+bx^{3}+cx^{2}+dx+e$$

where y stands for absorbance and x stands for wavelength.

**Figure 1:**
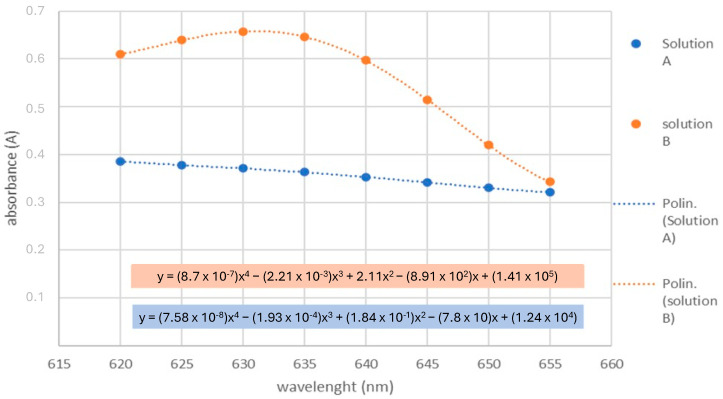
Dispersion graph with detailed regression equations for both solution A and solution B.

An example of this process is reported in Figure 1, which shows the sector of absorbance spectrum obtained with this method, complying with previously reported absorbance spectra for MetHb at ~645 nm [14,18,30].

The first derivative of the interpolating equation (Equation (1)) was then obtained with a specific algorithm. Namely, the algorithm took the a, b, c, and d coefficients, multiplied them by the exponent of the x associated with them, and used them to make the first derivative equation as follows:

(2) $$y\prime =4ax^{3}+3bx^{2}+2cx+d$$

The value of the first derivative was then calculated at x = 645 nm for both solutions.

The chosen wavelength (645 nm) [30] is the point of maximum steepness for the absorbance spectrum of MetHb, while the absorbance spectra for the other Hb forms are at their minimum with an almost horizontal trend. Since the first derivative of a function represents its steepness, this is the point of maximum difference between the steepness of MetHb’s spectra and other Hb forms’ spectra. Therefore, the first derivative was calculated at this point both for solution A and solution B to guarantee the maximum sensibility possible:

(3) $$A_{value}={\left[\frac{d^{\prime }Abs_{solution\;A}}{d\lambda }\right]}_{645}$$

(4) $$B_{value}={\left[\frac{d^{\prime }Abs_{solution\;B}}{d\lambda }\right]}_{645}$$

Since the graph obtained from solution A comes from a linear combination of the graphs of all Hb forms present in the sample, and since MetHb is the only Hb form that concurs in the increase of steepness, it is possible to calculate the MetHb percentage (%MetHb) in the sample by the following equation:

(5) $$\%MetHb=\frac{A_{value}}{B_{value}}*100\%$$

### 2.7. Statistical Analysis

The normal distribution of %MetHb values per year was preliminarily assessed through graphical analysis (histograms and probability plots). Skewness and Kurtosis values were then evaluated, and the data were checked for normality using analytical (Kolmogorov–Smirnov and Shapiro–Wilk’s tests). Because the normality of the data could not be assumed, a Kruskal–Wallis H test was ultimately performed to assess between-group differences in %MetHb values by year.

Additionally, the Jonckheere–Terpstra test was employed to evaluate trends across the groups over time. All statistical analyses were performed using IBM SPSS Statistics version 29 (IBM Corporation, Armonk, NY, USA), with two-tailed *p*-values < 0.05 considered statistically significant.

## 3. Results

The results for each sample are shown in Table 1. As reported, some cases (12, 17, 24, and 25) show a %MetHb higher than 70% even though only one month or less had elapsed since sample collection. It may seem unlikely that such a high concentration in such a short time may be due only to storage conditions. For samples 12, 20, and 21, no evidence that may justify an ante-mortem increase in MetHb was found. On the other hand, in one case (sample 17), the cause of death was a car accident that occurred under the influence of cocaine. As reported in the literature [31,32], cocaine may cause increased methemoglobinemia, probably due to the presence of lidocaine in the cutting agents. Therefore, this might be the cause for the increased value of %MetHb in this case.

**Figure 2:**
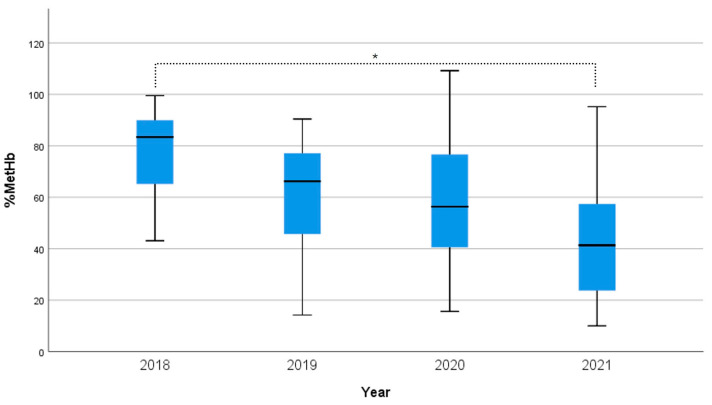
Distribution of MetHb percentual concentration in blood samples collected over four years (2018–2021) during judicial autopsies and stored in the toxicology laboratory. The years indicate the time of sampling and the start of storage. Boxes are the median concentrations and interquartile range; whiskers are 5% and 95% percentiles. * Significant difference between 2018 and 2021 groups (Kruskal–Wallis H test; *p* < 0.001).

The distribution of %MetHb concentrations in blood samples considered in our retrospective analysis, collected over four years (2018–2021) during judicial autopsies, is presented in Figure 2.

A significant overall effect was observed across the years (H(3) = 24.94, *p* < 0.001). Pairwise comparisons with adjusted *p*-values revealed a significant difference between 2018 and 2021 (*p* < 0.001). Furthermore, %MetHb was significantly influenced by the year, as the Jonckheere–Terpstra test (J = 901, z = 4.98, *p* < 0.001) indicated a significant descending trend in the medians over the years.

Interestingly, at a visual observation, all samples from 2018, 2019, and 2020 showed the typical “brown chocolate” color described in the cases of a high percentage of MetHb. The same colorimetric modification occurred for samples 12 and 17 from 2021, while the difference was not evident for other samples from the same period.

**Figure 3:**
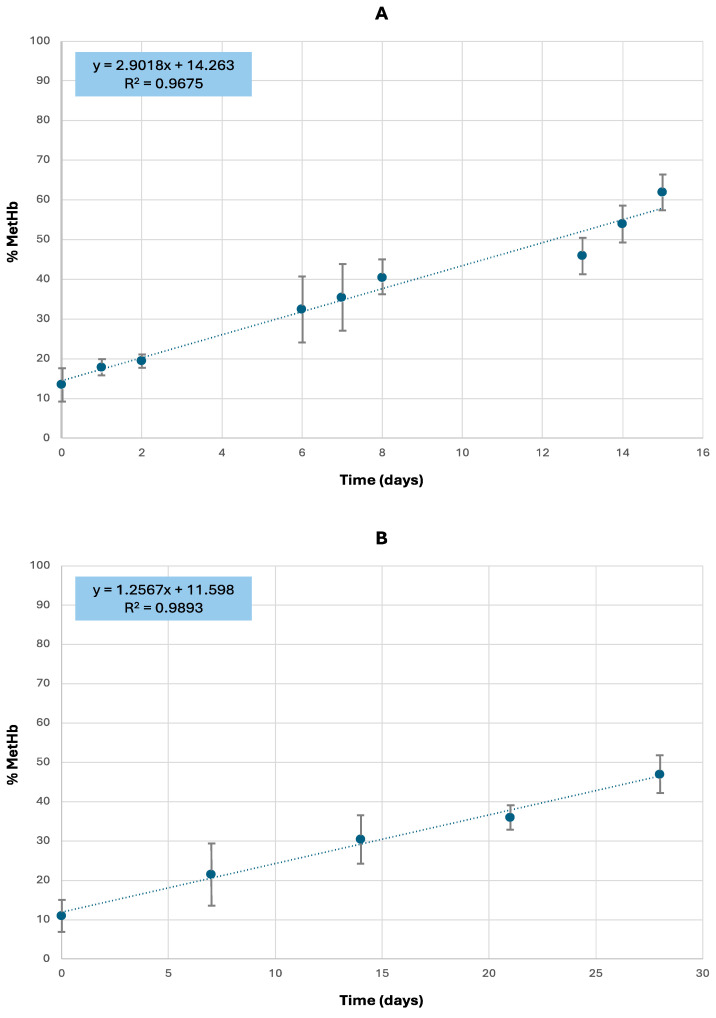
Increase of MetHb concentration over time for (**A**) fresh samples unfrozen in short three-day analytical periods (days 0, 1, 2, 6, 7, 8, 13, 14, and 15), and (**B**) fresh samples unfrozen every week.

The second stage of the study involved analyzing the percentage increase in MetHb over a short period. For this stage, samples collected during judicial autopsies and processed immediately were used. These samples were analyzed right after collection to establish the baseline MetHb concentration (%MetHb at time zero). Following this, the samples were aliquoted into two portions and frozen for further analysis. They were analyzed in short three-day analytical periods, and every week (i.e., every seventh day), respectively. The MetHb concentration was plotted against time, and a linear least-square regression was done to interpolate the obtained data. The results are presented in Figure 3.

A mean %MetHb value for all samples analyzed before the first freezing was calculated and resulted in 14.6 ± 2.2%. The rate of %MetHb increase was higher for samples subjected to frequent freeze–thaw cycles, showing a daily increase of 2.9%, compared to samples thawed weekly, which exhibited a daily increase of 1.3%.

## 4. Discussion

Our study aimed to evaluate the spontaneous formation of MetHb over time under critical storage conditions of blood samples. The results suggest various considerations about MetHb formation threshold, and a correlation between MetHb concentration and time since sampling.

Referring to MetHb formation, some samples showed MetHb levels ranging from 95 to 110% (cases 17, 48, 50, 82, 83, and 92). The data obtained indicate that a maximum threshold for MetHb is not applicable when classical storage methods are used.

Secondly, the post-mortem formation of MetHb occurs rapidly and appears to be influenced by the time since sampling and the number of freeze–thaw cycles experienced by the samples.

Specifically, the analysis of fresh samples over time confirms a linear relationship between MetHb formation and the elapsed time since collection and analysis. Furthermore, this study corroborates previous research [26,28] by finding a strong correlation between MetHb concentration and time since sampling. On the other hand, it is necessary to consider that in the “fresh” samples even the %MetHb found at the first analysis can be significantly higher than the physiological MetHb values, normally 1.5–2% [33]. This increase in %MetHb may be related to the post-mortem interval before sample collection, which generally is 48–96 h. These findings suggest that blood degradation begins early in the post-mortem period, and MetHb values around 15% at the time of autopsy should be considered normal, despite exceeding physiological levels.

Additionally, the analysis of fresh samples over time confirms that the freezing and thawing process impacts the rate of MetHb formation. In our study, the rate of MetHb increase was higher for samples subjected to frequent freeze–thaw cycles, showing a daily gain of 2.9%, compared to samples thawed weekly, which exhibited a daily increase of 1.3%. This evidence aligns with findings previously reported in the literature by other authors [26,28], emphasizing the critical role of temperature and storage conditions in explaining potential MetHb variations. Specifically, it has been reported that Hb conversion into MetHb via autoxidation is lower at temperatures below −20 °C, with samples remaining practically stable at −80 °C or −196 °C. Therefore, storage with cryoprotectants at −30 °C, or without any additives at −80 °C or −196 °C, is suitable for the long-term preservation of blood samples from autopsy cadavers for MetHb determination. Other studies have demonstrated that storage temperatures ranging from −12 to −20 °C present additional challenges and should be avoided, as MetHb levels in frozen–thawed specimens have been observed to increase over time, from 1.8% at six hours to 10.9% after six days [34,35].

In these cases, it is also necessary to consider the possible effects played on the samples by enzymatic reduction of MetHb to Hb by intraerythrocytic MetHb reductase, which could continue after death and is thermally deactivated, and MetHb decrease occurring in hemolyzed post-mortem blood due to bacterial contamination [35,36]. These considerations, together with the evidence found in the present study and the literature, suggest that post-mortem MetHb should not be measured on contaminated samples, and the analysis should preferably be performed rapidly after collection or, in any case, on adequately preserved samples.

Possible future perspectives may focus on the comparison of MetHb concentration calculated through this analytical method with data obtained from similar cases with more precise and advanced methods, in order to assess the possible existence of bias and ensure the reliability of these preliminary statements. A comparison with other methods should also help to estimate the possible overestimation of %MetHb due to basal increased values of sulfhemoglobin in post-mortem samples determined by putrefaction before sample collection. Another interesting perspective derives from samples not showing any MetHg increase in the absence of preservatives over time. The reason for this unusual occurrence should be investigated in depth to find a possible scientific reason.

## 5. Conclusions

The occurrence of suicidal consumption of nitrite and nitrate salts has been increasing all over the world, as reported in the forensic scenario [6,7,9,12,37,38,39,40,41,42,43,44,45]. A tempestive analysis, performed as soon as the samples are received in the laboratory, is crucial to obtain the maximum reliability and diagnostic values from data when MetHb quantitation is necessary. In these cases, it should be also considered that MetHb formation could be dependent on time passed since samples collection and the number of freezing/thaw cycles, if stabilizers are not used.

The present derivative spectrophotometry method provides an indirect evaluation for the analysis of nitrites/nitrates and should be, therefore, considered more as an initial analytical screening procedure than a confirmation method. However, it could be crucial in identifying those cases in which an increased methemoglobinemia is worthy of further study. Since the described procedure is fast, user-friendly, and cost-effective, it may, therefore, be advisable to introduce such an orientation and preliminary analysis, applicable in routine procedures for unexpected deaths in teenagers and young adults, even when no circumstantial data or strong evidence suggests possible ingestion of nitrites/nitrates. This should help to prevent a reanalysis of samples after long periods, when data from the toxicological assessment may also be unreliable due to sample storage conditions and consequent blood degradation.

## Figures and Tables

**Table 1:** MetHb percentual concentration for the analyzed samples (n = 95), the year in which each sample was collected, the storage time in the number of months or days, and the cause of death. For all samples, peripheral blood was used for the analysis.

Case	%MetHb	Year	Storage Time (Days)	Cause of Death	Case	%MetHb	Year	Storage Time (Days)	Cause of Death
1	15.3	2021	10	Heart attack	48	104.3	2020	269	Suicide (hanging)
2	15.7	2021	20	Benzodiazepines intoxication	49	15.6	2020	274	Car accident
3	11.1	2021	8	Car accident	50	109.2	2020	243	Homicide
4	26.0	2021	16	Suicide (hanging)	51	87.5	2020	235	Heroin intoxication
5	10.0	2021	17	Homicide	52	69.7	2020	239	Car accident
6	12.8	2021	10	Heroin intoxication	53	18.5	2020	229	Suicide (hanging)
7	16.2	2021	11	Heroin intoxication	54	59.1	2020	222	Suicide (shooting)
8	21.1	2021	15	Fall from stairs	55	58.1	2020	225	Car accident
9	21.4	2021	16	Car accident	56	92.4	2020	226	Precipitation
10	30.4	2021	24	Heart attack	57	53.7	2019	728	Suicide (shooting)
11	42.0	2021	10	Suicide (shooting)	58	60.1	2019	831	Homicide
12	84.1	2021	10	Heroin intoxication	59	76.1	2019	598	Car accident
13	54.1	2021	25	Precipitation	60	48.4	2019	684	Heroin intoxication
14	38.7	2021	26	Meth/Heroin Intoxication	61	43.0	2019	665	Heart attack
15	32.5	2021	24	Car accident	62	68.8	2019	722	Heart attack
16	29.2	2021	30	Homicide	63	89.7	2019	615	Methadone intoxication
17	95.2	2021	24	Car accident Cocaine use	64	19.5	2019	698	Car accident
18	56.8	2021	26	Suicide (hanging)	65	72.2	2019	607	Homicide
19	39.5	2021	25	Diazepam overdose	66	87.9	2019	664	Car accident
20	73.7	2021	29	Car accident	67	78.5	2019	630	Heart attack
21	72.5	2021	30	Car accident	68	39.6	2019	726	Benzodiazepines intoxication
22	28.2	2021	22	Precipitation	69	66.2	2019	707	Fall from ladder
23	12.7	2021	21	Fall from stairs	70	77.7	2019	692	Methadone intoxication
24	40.7	2021	25	Heroine intoxication	71	43.0	2019	683	Car accident
25	62.0	2021	30	Mechanical suffocation	72	14.2	2019	694	Meth/Heroine intoxication
26	38.8	2021	42	Suicide (shooting)	73	55.2	2019	669	Suicide (hanging)
27	48.2	2021	45	Heart attack	74	76.5	2019	640	Heart attack
28	68.1	2021	55	Heart attack	75	90.4	2019	635	Benzodiazepines intoxication
29	63.2	2021	58	Methadone intoxication	76	88.8	2018	977	Car accident
30	57.3	2021	66	Heroin intoxication	77	50.8	2018	1069	Heart attack
31	49.6	2021	65	Lorazepam overdose	78	89.3	2018	1082	Suicide (shooting)
32	70.2	2021	70	Car accident	79	89.9	2018	972	Car accident
33	51.4	2021	78	Homicide	80	83.5	2018	1128	Methadone intoxication
34	53.5	2021	77	Car accident	81	70.2	2018	1080	Precipitation
35	57.5	2021	92	Precipitation	82	97.7	2018	1138	Methadone intoxication
36	51.2	2021	89	Suicide (hanging)	83	98.4	2018	1087	Suicide (hanging)
37	82.1	2020	99	Methadone intoxication	84	93.2	2018	1246	Car accident
38	54.0	2020	95	Benzodiazepines intoxication	85	65.1	2018	1231	Heroin intoxication
39	44.3	2020	355	Heroin intoxication	86	65.2	2018	1292	Heart attack
40	71.2	2020	348	Suicide (shooting)	87	71.5	2018	1143	Car accident
41	27.6	2020	341	Car accident	88	90.0	2018	1167	Suicide (shooting)
42	49.1	2020	340	Heart attack	89	43.1	2018	1170	Car accident
43	60.6	2020	314	Heroin intoxication	90	52.0	2018	1168	Precipitation
44	28.7	2020	300	Suicide (hanging)	91	63.4	2018	1156	Heart attack
45	53.2	2020	308	Homicide	92	99.5	2018	1219	Suicide (hanging)
46	36.7	2020	284	Methadone intoxication	93	83.3	2018	1196	Meth/Heroine intoxication
47	54.6	2020	289	Car accident	94	76.6	2018	1204	Car accident
					95	83.5	2018	1321	Fall from stairs

## Data Availability

The data presented in this study are available on request from the corresponding authors.
